# Selection on Alleles Affecting Human Longevity and Late-Life Disease: The Example of Apolipoprotein E

**DOI:** 10.1371/journal.pone.0010022

**Published:** 2010-04-02

**Authors:** Fotios Drenos, Thomas B. L. Kirkwood

**Affiliations:** Institute for Ageing and Health, Newcastle University, Newcastle Upon Tyne, Tyne, United Kingdom; University of Washington, United States of America

## Abstract

It is often claimed that genes affecting health in old age, such as cardiovascular and Alzheimer diseases, are beyond the reach of natural selection. We show in a simulation study based on known genetic (apolipoprotein E) and non-genetic risk factors (gender, diet, smoking, alcohol, exercise) that, because there is a statistical distribution of ages at which these genes exert their influence on morbidity and mortality, the effects of selection are in fact non-negligible. A gradual increase with each generation of the *ε2* and *ε3* alleles of the gene at the expense of the *ε4* allele was predicted from the model. The *ε2* allele frequency was found to increase slightly more rapidly than that for *ε3*, although there was no statistically significant difference between the two. Our result may explain the recent evolutionary history of the epsilon 2, 3 and 4 alleles of the apolipoprotein E gene and has wider relevance for genes affecting human longevity.

## Introduction

Evidence points towards the existence of a strong heritable component of human longevity [1], [2]. Around a quarter to a third of the variability of lifespan can be attributed to the action of genes [3], [4], [5], [6], [7]. One of the best examples of a gene affecting survival in old age is the apolipoprotein E gene *APOE*. Situated on human chromosome 19 at locus 19q13.32 (http://genome.ucsc.edu/, assembly (hg18)), the *APOE* gene is 3.6 kb long containing four exons and coding for a 317 amino-acid polypeptide that after cleavage gives rise to a 299 amino-acid long mature protein [8], [9]. Apolipoprotein E (APO E) is a member of a diverse family of carrier proteins specializing in lipoprotein particle formation, secretion, transport, binding and metabolism [10]. APO E is synthesized in many different regions of the body such as the liver, brain (primarily astrocytes), skin, macrophages and steroidogenic organs [11].

APO E has three major and more than thirty minor isoforms, the latter being mostly linked with disease. The three common alleles are epsilon (*ε*) 2, 3 and 4 producing three homozygous (*ε2*/*ε2*, *ε3*/*ε3* and *ε4*/*ε4*) and three heterozygous (*ε2*/*ε3*, *ε3*/*ε4* and *ε2*/*ε4*) genotypes [12]. The difference between the alleles lies at two amino acid residues 112 and 158; with the most common allele *ε3* having cysteine and arginine at these residues, respectively, while *ε2* has cysteine and *ε4* arginine at both locations [9]. Carriers of different alleles show differences in the incidence of coronary artery disease (CAD), peripheral atherosclerosis, Alzheimer disease, possibly stroke and even ability to recover from trauma [11], [13], [14], [15], [16]. The underlying mechanism for the action of the gene involves an inter-domain interaction between the amino- and carboxyl- terminals of the protein, producing isoform-specific lipoprotein preferences such that E2 and E3 protein isoforms bind preferentially to HDL (the “good” cholesterol), while the E4 isoform shows a preference for VLDL [17]. The APO E2 isoform is defective in binding the LDL receptor, although it retains its ability to interact with LDL-receptor-related protein and other related receptors [11]. A small number of *ε2* homozygotes suffer from a condition known as type III hyperlipoproteinemia, characterized by accumulation of cholesterol-rich remnant lipoproteins due to incomplete catabolism of chylomicrons and VLDL, leading to premature atherosclerosis. The fact that although 90% of the patients are *ε2* homozygotes, but only 5% of the *ε2* homozygotes suffer from the condition, suggests that further factors are required for the phenotypic expression of the condition [18], [19].

Generally in Caucasian populations survival to advanced age is more likely for carriers of the *ε2* allele than for *ε3* homozygotes and less likely for carriers of the *ε4* allele [20], [21], [22], [23], [24]. The *ε4* allele is strongly associated with increased risk of coronary heart disease (CHD) [14], [25], [26], [27], [28], while for the *ε2* allele there is some, though weaker, evidence for a protective effect [29], [30]. The APOE polymorphism, has also been implicated in Alzheimer's disease (AD), which is the commonest form of senile dementia [31], [32]. With the *ε4* allele shown, in a number of studies, to be associated with both familial and sporadic forms of the disease, causing higher incidence and earlier age of onset, and affecting its pathology and rate of progression [33], [34]. Normal age-associated cognitive decline, and mild cognitive impairment and the risk of its conversion to AD have also been repeatedly associated with the presence of the *ε4* allele [35].

One of the major ideas in the evolutionary theory of ageing [36] is the suggestion that, because the force of natural selection declines with age, alleles with deleterious effects seen only at older ages can reach higher frequencies than those that have their effects earlier in life [37]. Therefore, if a gene exerts an effect only after the end of the reproductive phase of the lifespan it has been thought unlikely that it could have been subject to significant direct selection pressure, and this would seem to be the case for genes affecting late-life diseases such as AD and CHD [38], [39]. Nevertheless, the worldwide abundance of *ε3*, as compared to *ε4* which, from studies in non-human primates appears to be the ancestral allele [40], [41], suggests that selection has acted upon these alleles. Here, we use a simulation approach based on the known risk factors for cardiovascular disease (CVD) to examine the hypothesis that the evolution of the *APOE* gene was, and still is, driven by its role in lipid metabolism and its subsequent effects on health.

## Methods

### Risk factors

The selection of modifiable risk factors to include in the simulation was based on current knowledge of lifestyle parameters affecting cardiovascular disease (CVD) while keeping in mind the need for independence between the risk factors, simplicity and availability of data. There are two categories of risk factors associated with CVD: *non-modifiable* risk factors, such as *APOE* genotype, and gender, and *modifiable* risk factors, such as smoking, unhealthy diet, lack of physical activity and high alcohol consumption [42]. For the non-modifiable risk factors, the estimated genotype relative risk used was from Gerdes et al [29], while the gender difference in CVD was based on the results of Panagiotakos et al [43]. To quantify the effect of diet, we used the five clusters described by Millen et al [44] in the Framingham study (Heart Healthy, Light Eating, Wine and Moderate Eating, High Fat, and Empty Calorie) [44], [45]. For alcohol, smoking and exercise, we used the risk estimates from Stampfer et al [46].

### Genotype-environment interaction

A number of studies found evidence of interaction between the *APOE* genotype and each of the modifiable risk factors considered in the model (diet, [9], [47], [48], [49]; alcohol [50], [51], [52]; smoking [53], [54]; and exercise [55]), although the results are sometimes conflicting. Importantly, their precise interaction in quantitative terms remains unknown and will ultimately require a series of studies in large populations before the levels of genotype-environment interactions can be estimated with adequate precision.

Here we use the association of *APOE* and diet to illustrate the principles used to account for the unknown interaction terms. Our first assumption was that, except *APOE*, the risk factors considered were independent of each other. Then we assumed that the relative risk weighted average of the risk factor, for all APOE alleles, was approximately 1, i.e. equal to the reference *ε3ε3* genotype. According to the Odds Ratios (OR) given in Gerdes et al [29] the weighted average is (*ε2* OR×*ε2* freq+*ε3ε3* OR×*ε3ε3* freq+*ε4* OR×*ε4* freq; 0.88×0.11+1×0.71+1.1×0.18 = 1.005). Treating both variables as ordinal, we can fit a model between the two factors; in this case a quadratic model gave the best fit to the data. Assuming that all genotypes will have the same OR at some hypothetical level of the modifiable risk factor and considering that the overall OR between the *APOE* genotypes is dependent on their weighted difference for each category of diet intake, making use of the correspondence of the overall weighted mean to the *ε3ε3* genotype, we can work out the beta coefficient for each of the other *APOE* genotypes. Using similar techniques we computed the interaction components for all of the remaining risk factors.

### Calculating risk

Despite OR overestimating risk for common diseases such as CVD [56], [57], many researchers do use it to report their results. To approximate the relative risk (RR) from the OR we used the very popular methodology of Zhang and Yu [58] as being both the simplest and the one that requires the least information for the dataset used. Despite the method's slight inaccuracy in calculating confidence intervals and its inability to account for confounding factors, its balance between simplicity and precision make it an especially useful tool [56], [57].

The total risk of an individual could be calculated as the product of the gender relative risk and all four modifiable risk factors, corresponding to the individual's specific genotype. We used a proportional hazard model to estimate the mortality schedule of an individual having relative risk *R* at a specific age *x*. According to this, if *μ_1_*(*x*) is the hazard of death of genotype 1 at age *x*, and *μ_2_*(*x*) is the hazard of death of a genotype 2 at the same age *x*, then *μ_1_*(*x*) = *R*×*μ_2_*(*x*), where *R* expresses the relative risk [29].

### Simulating evolutionary change

Our simulated populations comprised individuals with randomly assigned genotype, gender and lifestyle parameters from a typical western population. The random draws were done using the MT19937 pseudorandom generator, a variation of the “Mersenne Twister” generator (GSL Reference Manual). Equal number of males and females were generated and subsequently paired as couples randomly. These couples reproduced to age 50 or until either of the individuals died. The reproductive schedule was the same as what is found in contemporary western populations, with a series of binomials distributions describing the chance of a successful birth for each year of life and the probability decreasing with increasing adult age. The age of death was obtained from a Gumbell distribution based on demographic tables for the UK (Office for National Statistics) and a relative risk corresponding to the life style parameters of the simulated individual. Each offspring was given a genotype based on the genotypes of its parents, and all the produced genotypes were pooled into a matrix constituting the initial conditions for the next generation. The process was repeated as many times as the number of generations required. The entire simulation was written in C++ and performed using a 16 CPUs Unix cluster.

### Statistical analysis of the results

The Mathematica 4.1 package was used to collect analyse the output of the simulations. To minimize random variation of the results, a set of 60 runs, each comprising 200,000 individuals followed for 50 generations, was used. We summarized the data calculating the mean and standard deviation of all 60 runs for each generation and each allele. A random walk in one dimension was used to obtain the average change per generation, assuming that the change of the allele frequencies was linear and could be described by a simple equation. As long as the alleles do not have a pleiotropic effect balancing the selection for or against them, their frequencies in a stable environment were expected to increase until they were fixed or became extinct in the population. A simple equation describing the change could be written as:
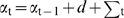
(1)where *α_t_* is the frequency at time or generation *t*, *α_t-1_* is frequency in the previous generation, *d* is the change between two consecutive points in the series and Σ*_t_* is random noise normally distributed. In order to calculate *d* we estimated a *y_jt_* such that:
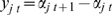


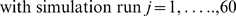


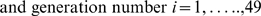
returning a matrix of 49×60 elements. From this, the mean *d* and its 95% confidence intervals (95% CIs) could be calculated. If the 95% CIs are positive then there is evidence for a significant increase of the allele frequency with each generation, while if negative the allele goes towards extinction. In the case that 0 is included within the confidence interval, the change of frequency cannot be considered statistically significant and selection is either extremely weak or due to chance. The data were also transferred into Minitab statistical software to test for statistical significance between and within different variants of the simulation.

## Results

**Figure 1:**
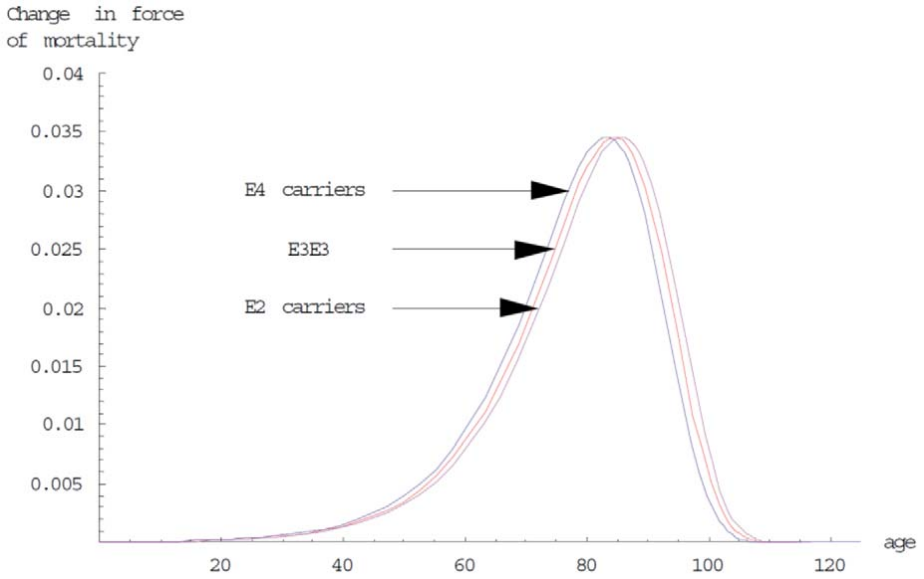
Differences in the distribution of mortality for the three *APOE* genotype classes. These are estimated using a proportional hazard model as described in the methods. The *ε2* carriers have an OR of 0.9 and survive a year more than the *ε3ε3* carriers. In contrast, *ε4* carriers have a decreased lifespan of 1.2 years due to an OR of 1.13 compared to the reference genotype.

**Figure 2:**
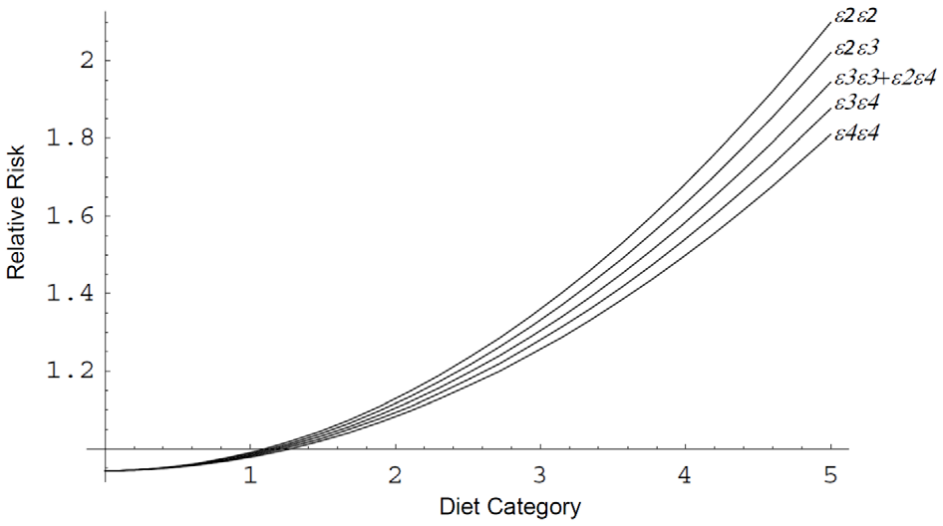
Inferred interaction between *APOE* genotype and diet. Diet categories are considered on an integer scale from 1 to 5 with Heart Healthy, Light Eating, Wine and Moderate Eating, High Fat, and Empty Calorie clusters [44], [45]. We assumed that for a hypothetical diet category 0 there is no distinction in risk between the genotypes.

**Figure 3:**
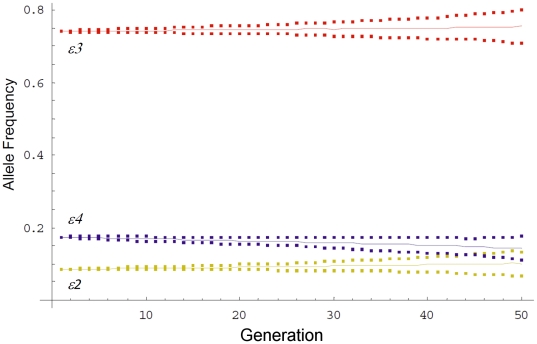
Change in frequency of the three *APOE* alleles with each generation. The long dash line represents the *ε3* allele, the solid line the *ε4* and the short dash line the *ε2*. The squares, stars and triangles points represent the 95% Confidence Intervals of the *ε3*, *ε4* and *ε2* frequencies respectively.

Starting with *APOE* genotype frequencies of 1.7% *ε2ε2*, 11.6% *ε2ε3*, 55.8% *ε3ε3*, 1.9% *ε2ε4*, 25.1% *ε3ε4*, and 3.9% *ε4ε4* and modifiable risk factor distributions as reported in Millen et al [44] and Stampfer et al [46], and assuming the same population distribution between males and females, the mean life expectancy at birth for the simulated individual was 75.7 years with females having 5.4 years longer expected lifespan than males. The differences in mortality between *APOE* genotype classes as fitted in the model can be seen in Figure 1, with *ε2* carriers living 1 (OR 0.9) year more, and *ε4* carriers living 1.2 (OR 1.13) years less, than the *ε3ε3* reference genotype. In figure 2, the curves obtained when the interaction between genotype and diet was considered can be seen, while table 1 has the calculated relative risk for each combination of diet and *APOE* genotype. For the force of natural selection on the *APOE* gene, we observed a positive selection for the *ε2* (mean frequency change per generation 3.352×10^−4^, 95% CI 2.661×10^−4^ to 4.043×10^−4^) and *ε3* (mean 2.785×10^−4^ 95% CI 1.809×10^−4^ to 3.761×10^−4^) alleles against *ε4* (mean −6.137×10^−4^ 95% CI −6.946×10^−4^ to −5.327×10^−4^), with the *ε2* frequency increasing slightly more rapidly than that for *ε3*, although there was no significant difference between them (Figure 3).

**Table 1:** Calculated relative risk for each combination of diet category as described by Miller et al (2001) and *APOE* genotype.

*APOE* genotype	22	23	33	34	44
**Hypothetical diet 0**	0.944	0.944	0.944	0.944	0.944
**Heart Healthy**	0.979	0.981	0.984	0.987	0.990
**Light Eating**	1.083	1.093	1.104	1.116	1.129
**Wine and Moderate Eating**	1.256	1.280	1.304	1.332	1.360
**High Fat**	1.499	1.541	1.584	1.633	1.683
**Empty Calorie**	1.811	1.877	1.944	2.020	2.099

Considering that only a fraction of the population will suffer from *APOE* related diseases, a variant of the model with 30% of the total mortality attributed to CVD was also tested. Again, the results pointed to a positive selection of the *ε2* and *ε3* alleles (*ε2* mean 0.846×10^−4^, 95% CI 0.497×10^−4^ to 1.195×10^−4^; *ε3* mean 0.846×10^−4^, 95% CI 0.316×10^−4^ to 1.377×10^−4^) and a negative selection for the *ε4* allele (mean −1.692×10^−4^, 95% CI −2.140×10^−4^ to −1.244×10^−4^) which, as expected, was approximately one third of the change observed previously.

We performed a number of simulation runs with a variety of different starting conditions both to test the sensitivity of the model and analyse the likely changes under different lifestyle choices. The description of all the simulation variants used with their mean life expectancy at birth can be seen in table 2. The changes in the distribution of the modifiable risk factors explored, except the eradication of smoking, were relatively modest and considered possible to occur, if not already present in certain subgroups of western populations. It is important to note the unexpected decrease in mean lifespan when heavy alcohol consumption is lowered and the slight increase in longevity when non- and very light drinkers decrease. This effect is due to the U-shaped relationship between alcohol consumption and health and the re-arrangement of the population distribution across the categories of risk.

**Table 2:** The simulated average lifespan in years of each variant considered in the absence of non APO E related mortality.

Run	Parameters	Mean Lifespan
Default	Default	75.69
Default 2	Default values with 30% CVD associated mortality	75.69
Diet 1	Heart Healthy and Light Eating groups decreased by 20%	74.93
Diet 2	High Fat and Empty Calorie groups decreased by 20%	75.98
Alcohol 1	Light and non-drinkers decreased by 25%	75.97
Alcohol 2	Heavy drinkers decrease by 25%	75.54
Exercise 1	Entire population moderately active (2.5h/week)	76.19
Exercise 2	Most active groups decreased by 25%	75.41
Smoking 1	Non-smokers decreased by 25%	75.57
Smoking 2	No smokers in the population	82.95

The estimates for the mean frequency change for each *APOE* allele per generation, under all the different models considered assuming at the same time that only 30% of the population is affected by any *APOE* related diseases, are presented in table 3. As can be seen in the table, the selection against the *ε4* allele and the increase in the frequency of the *ε3* and *ε2* alleles are robust and remained significant under all the different versions of the simulation. A notable exception was the loss of significance for the increase of the *ε3* frequency when the effect of smoking is removed from the population (mean 0.259×10^−4^, 95% CI −0.057×10^−4^ to 0.574×10^−4^). The extreme change of removing all smokers in the population, while in the same time leaving the percentage of CVD associated mortality at 30%, reaches the statistical power limits of our current simulation to observe significance for the selection of the *ε3* allele in a population of 20,000. For such extreme cases, a corresponding change should also be made to the mortality schedule and the fraction of the population affected by that change.

**Table 3:** Mean change of the frequency of each allele for the variants of the model used together with the 95% CI of the standard error of the mean in each case.

Run	APO E allele	Mean change of allele frequency per generation	95% confidence intervals for the mean	
	ε2	0.0003352	0.0002661	0.0004043
Default	ε3	0.0002785	0.0001809	0.0003761
	ε4	−0.0006137	−0.0006946	−0.0005327
	ε2	0.0000846	0.0000497	0.0001195
Default 2	ε3	0.0000846	0.0000316	0.0001377
	ε4	−0.0001692	−0.0002140	−0.0001244
	ε2	0.0000900	0.0000547	0.0001252
Diet 1	ε3	0.0000899	0.0000360	0.0001438
	ε4	−0.0001798	−0.0002257	−0.0001340
	ε2	0.0000809	0.0000480	0.0001139
Diet 2	ε3	0.0000643	0.0000130	0.0001157
	ε4	−0.0001453	−0.0001905	−0.0001000
	ε2	0.0000836	0.0000524	0.0001148
Alcohol 1	ε3	0.0000818	0.0000326	0.0001309
	ε4	−0.0001654	−0.0002081	−0.0001226
	ε2	0.0000847	0.0000500	0.0001195
Alcohol 2	ε3	0.0000588	0.0000074	0.0001103
	ε4	−0.0001436	−0.0001880	−0.0000991
	ε2	0.0000895	0.0000555	0.0001235
Exercise 1	ε3	0.0000629	0.0000108	0.0001149
	ε4	−0.0001524	−0.0001959	−0.0001088
	ε2	0.0001088	0.0000749	0.0001427
Exercise 2	ε3	0.0000637	0.0000115	0.0001158
	ε4	−0.0001725	−0.0002171	−0.0001279
	ε2	0.0001283	0.0000897	0.0001668
Smoking 1	ε3	0.0000974	0.0000383	0.0001564
	ε4	−0.0002256	−0.0002766	−0.0001746
	ε2	0.0000406	0.0000205	0.0000607
Smoking 2	ε3	0.0000259	−0.0000057	0.0000574
	ε4	−0.0000665	−0.0000936	−0.0000393

## Discussion

We have shown that under certain environmental conditions, such as those often found in Western populations, the *APOE* gene is likely to be under the action of natural selection. According to the results obtained, the *ε2* and *ε3* alleles are increasing with each successive generation at the expense of the *ε4* allele, which is slowly being removed from the population. The selection against the *ε4* allele was found to be robust, despite changes to the initial conditions of the simulation. We failed to find any significant difference between the increase in frequency of the *ε2* and *ε3* alleles, denoting their very similar effects on survival. This accords with meta-analysis studies which have found it difficult to identify any statistically significant risk differences between the carriers of *ε2* allele and the reference *ε3ε3* genotype, unless a large number of cases is available, mainly due to the low frequency of *ε2* in the populations considered [15].

Despite the difficulty in observing and measuring the force of natural selection as revealed by change of allele frequencies in specific polymorphisms, especially in humans, alternative methods are available to obtain evidence for its action indirectly. *APOE* seems to be one of the least variable human genes studied, despite the average neutral mutation rate [59]. Comparing the chimpanzee and human *APOE* genes, revealed that *ε4* is probably the ancestral allele [59]. Indeed, most great apes carry only the *ε4* allele, although a change of arginine to threonine at position 61 gives this isoform an affinity similar to the human *ε3* [40], [60], [61]. It is unclear when the human *ε4* allele arose, but a comparison between coding changes in orthologous genes in five species (Mouse, Rat, Dog, Chimpanzee and Human) showed positive selection for *APOE* along the hominid lineage [62]. Using the haplotypes defining the three genotypes in a coalescent model, Fullerton et al [59] proposed that *ε3* diverged from the *ε4* haplotype around 200,000 years ago and that *ε3* is showing evidence of increasing in frequency, relative to *ε4*. Assuming a constant selective pressure and a generation time of ∼20 years, given an *ε3* frequency of around 0.75, a very simple calculation shows that the required increase of *ε3* per generation is 7.5×10^−5^, close to the order of magnitude predicted from the simulation for the current change of the allele frequency. Using a similar argument, we can further propose that since *ε2* and *ε3* alleles seem to be under a similar pressure from natural selection in our simulation and *ε2* has a lower frequency in human populations, *ε2* arose later than *ε3*. Again, analysis of *APOE* haplotypes using a reduced median network revealed that *ε2* was derived from the *ε3* allele within the last 80,000 years [59].

Assuming a simple and uniform selection pressure on *APOE* is rather over simplistic. Current theories suggest a two step evolution of the locus, one to explain the early evolution of the human alleles and a second step to account for their current global frequencies. Despite the very sparse evidence for both, and their rather conflicting views for the selection pressures applied, the proposed mechanisms are interesting and reveal the importance of *APOE* in human evolution. Finch and Stanford [63] and Finch and Sapolsky [60] suggested that *APOE* is a “meat adaptive” gene that permitted increased consumption of animal tissue during hominid evolution while conferring resistance to the associated risks such as hypercholesterolemia and infections, allowing for the extension of human lifespan. In their view, this increase in meat eating provided humans with a solution to seasonal deficits of micronutrients. In addition, relatively safe consumption of nutrient dense meat is believed to have lead to an increase of body size, without restricting activity, and conferred the extra energy required for the cerebral expansion of early humans [64], [65]. On the other hand, the frequencies of the three common alleles vary widely between populations of different ethnic backgrounds. In general *ε3* is globally the most common allele, with Africans having the lowest allele frequency (≤70%) compared to Caucasians (70–80%) and East Asians (≥80%) [66]. In Europe, and between populations of Caucasian descent, there is a north-to-south gradient of decreasing *ε4* frequency opposite to the spread of agriculture [67], [68]. This has led Corbo and Scacchi [69] to suggest that *APOE* is a thrifty gene, with the *ε4* allele being advantageous under seasonal periods of starvation, due to its elevating effect of cholesterol which would otherwise be too low, but detrimental in areas where carbohydrates were readily available, such as those with a long history of agriculture, thus the north-to-south pattern observed in Europe. Doubts have been raised about the lack of seasonal starvation in food producing societies [70], while the hypothesised selection for *ε4* in pre-agricultural times is opposite to the one suggested by Finch and Sapolsky [60], [63].

Although it is clear that variation in the *APOE* locus has been, and probably still is, under the control of selection, the fact that its main effects are beyond the reproductive lifespan of humans, has posed an interesting puzzle for the mechanisms through which selection is applied. Finch and Sapolsky [60], [71] suggested that the spread of *ε3* in human populations is due to the effect of a mother's late survival on the fitness of her offspring. The grand-mother hypothesis, as it is known, is centred on the idea that the mother-child food sharing seen among hunter-gatherers may allow post-reproductive grandmothers to enhance their daughters' fertility, thus elevating their own fitness and increasing the selection for long postmenopausal lifespan [72], [73], [74]. Finch and Sapolsky [60], [71] argued that this evolutionary advantage will have caused selection for an *APOE* allele that will have delayed neuropathology and mortality even if its effects are evident later in life (for counterarguments see [75], [76]). Charlesworth [77], on the other hand, proposed that *APOE* is an example of balanced polymorphism with the variation in the locus maintained due to the antagonistic effects of the alleles, where increased late life risk is offset by advantages in younger ages, in accordance with the antagonistic pleiotropy theory of ageing. Martin [76] extended the idea of antagonistic pleiotropy for *APOE* suggesting that the *ε4* allele could be advantageous in cases of infections with pathogens requiring host lipids for survival. Parasites like *Trypanosome brucei* not able to carry out de novo liposynthesis may depend upon host LDL for acquisition of lipids, with the high affinity of *ε4* for some lipids actually hindering their uptake by the pathogen. Our model indicates that, although these hypotheses might work at a secondary level, they are not strictly necessary to explain the selection of *APOE* alleles. The *direct* effect of *APOE* on the mortality or morbidity of the population is sufficient, at least in contemporary Western populations, to produce a selection differential between the three alleles. The simulation predicts that the *ε3* and *ε2* alleles are driven towards fixation, a conclusion supported by the Fullerton et al [59] observations for the mutation at site 3937, characterising the *ε3* allele.

This simulation study is considering the pressure of natural selection on a population with demographics equal, or similar, to what is found in contemporary populations. It will be wrong to extrapolate our results in order to interpret the early evolution of *APOE*, since modern populations experience a very different environment compared to our early ancestors. Nevertheless, our basic conclusion that certain genetic polymorphisms, such as *APOE*, exhibiting their main effect later in life can still be under the action of natural selection, still holds. Interestingly, analysis of a Danish cohort of individual born between 1895 and 1899, showed that carriers of the ε4 allele had increased early mortality compared to those carrying the ε2 allele as suggested by our simulation [78]. It is currently unclear how far back our results can be considered as valid, but given the early age (>35 years of age) that the *APOE* effects on mortality can be seen in the Danish cohort, we believe that this can extend in our recent history.

In our simulation we cannot account for any pleiotropic effects of *APOE*. We described the effects of the gene on lifespan only through its relation to elevated risk for CVD. *APOE* has a much wider role than just lipid metabolism, including immunoregulation and susceptibility to infections [60], [79], [80], [81]. We chose not to include these effects within the simulation since reliable quantitative data concerning their impact on lifespan do not exist. *APOE*, though its role in steroidogenesis, has also been shown to influence fertility in pre-industrial populations [82], old Italians (>80 years of age) [83] and North European males [84], with the *ε2* carriers having less children. The small number of *ε2* subjects together with the many cultural and socioeconomic factors affecting current human reproduction makes it difficult to say if this effect is indeed real and relevant in the model used. A limitation of our model was the lack of data for the interaction between the *APOE* genotypes and each of the modifiable risk factors considered, which forced us to use a very approximate method to calculate the unknown interactions. Further epidemiological work is required to elucidate the precise quantitative relationship of the *APOE* gene with the parameters of the model so that the predictions can be made more accurate. While, for simplicity, we assumed that the rest of the risk factors are independent of each other, although it has been recognised that these tend to cluster, to some degree, in individuals of high risk [85].

Despite some limitations, our results indicate that although the main effect of *APOE* is seen after the end of the reproductive lifespan of humans, the relatively rare events of early mortality or morbidity are enough to produce selection against the *ε4* allele. Considering these findings in the light of the evolutionary theory of ageing and longevity, particularly as regards the ‘mutation accumulation’ of late acting deleterious alleles, we can identify an important perspective on this concept. Since most longevity-associated genes, such as *APOE*, will cause a distribution around a mean for the corresponding mortality, evolution will rarely, if ever, manage to push deleterious gene action entirely beyond the reach of selection, although as envisaged by Medawar there will be a continual selection pressure to postpone such action to later and later ages. The gradual postponement of the detrimental gene effect will take place simultaneously in a wide array of mortality associated genes. Any single mortality distribution exposed significantly more often to the action of selection will be moved towards older ages faster, until it reaches a balance with all the other detrimental genotypes. This process will continue to drive the longevity of the organism until the age-related mortality becomes non-significant in relation to the age-independent mortality. In this respect the evolution of longevity, although based on selection of individual genes, is more of an evolutionarily coordinated transfiguration of the whole genome to achieve the maximum lifespan in the given conditions as hypothesized by Hamilton [38].
